# Perceived quality of AI-generated and human-written news: a cross-national experimental study among journalism students in China and Vietnam

**DOI:** 10.3389/fpsyg.2026.1886638

**Published:** 2026-09-23

**Authors:** Mingyi Yang, Yaqi Deng, Yuting Cheng

**Affiliations:** School of Journalism and Communication, Guangxi University, Nanning, China

**Keywords:** AI-generated news, cross-cultural comparison, generative artificial intelligence, news quality evaluation, power distance

## Abstract

**Introduction:**

Generative artificial intelligence is reshaping news production, yet how journalism students evaluate AI-generated content and whether cultural-psychological factors systematically shape these evaluations remain poorly understood, particularly in non-Western contexts.

**Methods:**

This study employed a 2 (Country: China/Vietnam) × 2 (Author Type: AI/Human) × 4 (Topic: Nature/Health/Society/Finance) mixed-design ANOVA with 140 journalism and communication students under blind conditions. Participants evaluated news quality across seven dimensions without knowing whether articles were AI-generated or human-written.

**Results:**

AI-generated news received significantly higher overall ratings than human-written news. This advantage was not uniform: a significant Country × Authorship interaction indicated that Chinese participants showed a substantially larger preference for AI-generated news than Vietnamese participants, whose evaluations of the two content types did not differ significantly; a significant Authorship × Topic interaction indicated that the AI advantage was more pronounced for Nature and Society news than for Health and Finance news; and the three-way interaction was not significant. AI-generated news outperformed human-written news specifically on structural dimensions (perceived headline relevance, coverage adequacy, readability, and punctuation and sentence structure) but not on perceived factual accuracy or source usage. Gender predicted evaluations of both news types without a significant authorship-specific asymmetry, whereas news interest and news trust predicted evaluations of human-written news more strongly than AI-generated news, a pattern confirmed by direct interaction tests.

**Discussion:**

These patterns are discussed in relation to culturally grounded orientations toward institutional authority and technology, including the authority heuristic and techno-optimism, although these constructs were inferred from country-level cultural indices rather than directly measured among individual participants. A post hoc textual analysis found that AI-generated articles were not lexically less diverse than human-written articles, which complicates homogenization-based accounts of the cross-national convergence observed in AI-generated news evaluations. These findings extend cross-cultural psychology by illustrating both the promise and the limits of using country as a proxy for culturally embedded psychological constructs in research on AI-generated content.

## 1 Introduction

The rapid development of generative artificial intelligence (GenAI) is transforming how textual content is produced, disseminated, and evaluated (Laupichler et al., 2025). Generative AI is now widely used across news organizations worldwide, from local newspapers and public broadcasters to large digital-native media outlets. A survey of journalism professionals across six continents found that 73.8% of respondents’ organizations have already applied generative AI to news production, with text creation being the primary use case (Dodds et al., 2026). As AI-generated content becomes more prevalent in newsrooms, how audiences perceive, evaluate, and place trust in AI-generated news has become a question of both theoretical and practical significance.

From a cultural psychological perspective, the evaluation of AI-generated news is not a purely technical judgment but a process deeply embedded in culturally shaped cognitive orientations. Individuals develop shared mental models within specific cultural environments. These models, which encompass self-construal, power distance beliefs, and uncertainty avoidance tendencies, systematically shape their attitudes toward emerging technologies, their trust in information sources, and their criteria for evaluating content quality. Hofstede’s cultural dimensions framework (Hofstede et al., 2010) provides a foundational analytical lens for understanding such cross-cultural variation, identifying systematic differences across societies in power distance, individualism-collectivism, masculinity-femininity, and long-term orientation that are directly relevant to how audiences process and evaluate algorithmically generated information. When people encounter content whose origin is ambiguous or unfamiliar, they draw on culturally acquired heuristics to assess its credibility, and these heuristics vary systematically across cultural contexts (Norenzayan et al., 2007).

Existing research has examined audience perceptions of AI-generated news from multiple perspectives, with a particular focus on credibility, trust, and perceived quality. Some studies suggest that psychological mechanisms such as algorithm aversion lead audiences to hold relatively negative attitudes toward AI-generated news, with people tending to distrust or avoid algorithmically produced content even when it is comparable in quality to human-written news (Zoizner et al., 2025). Others have found that readers often struggle to distinguish AI-generated news from human-written news during actual reading, and may even rate AI-generated news higher in credibility (Baptista et al., 2025; Jia, 2020; Clerwall, 2014; Wölker and Powell, 2021). Audience evaluations are also shaped by topic type, meaning they are not uniform across news categories (Graefe et al., 2018). The study most directly relevant to the present research is Baptista et al. (2025), who found that journalism students in Portugal and Spain rated ChatGPT-generated news higher in overall quality than journalist-written articles across all topic categories. Author disclosure adds another layer of complexity: when news is explicitly labeled as AI-generated, audiences tend to rate it lower even when the content is objectively comparable to human-written news (Wang and Huang, 2024; Jia et al., 2024), suggesting that intuitive judgments about AI rather than actual content quality drive credibility assessments when authorship is known.

Despite growing scholarly attention, systematic research on how audiences from non-Western cultural backgrounds evaluate AI-generated news remains scarce. As Henrich et al. (2010) have noted, psychological research has long relied disproportionately on samples from Western, educated, industrialized, rich, and democratic societies, substantially limiting the external validity of its theoretical conclusions. A comparative study involving American and Chinese users found that Chinese participants showed relatively greater acceptance of algorithmically generated news content, with the two groups diverging in their evaluations of quality, preference, and credibility (Zheng et al., 2018). Beyond individual factors, attitudes toward AI are shaped by cultural values, national media systems, and prior familiarity with the technology (Araujo et al., 2023). These findings suggest that culturally embedded psychological orientations systematically shape how audiences perceive and evaluate AI-generated content, yet this dimension remains underexplored in the existing literature.

China and Vietnam offer a theoretically productive comparative context for examining these cultural dynamics. Both societies share Confucian cultural heritage, collectivist social orientations, and party-directed media systems, positioning them as a culturally proximate yet internally differentiated pair that differs substantially from the Iberian samples studied by Baptista et al. (2025). At the same time, the two countries exhibit meaningful variation on key Hofstede cultural dimensions: China scores higher than Vietnam on both power distance (80 vs. 70) and long-term orientation (87 vs. 57), and is more masculine in orientation (66 vs. 40), While these national-level indices cannot be assumed to directly characterize individual participants’ psychological orientations, they suggest that Chinese and Vietnamese students may, on average, bring different evaluative criteria to bear on AI-generated content, a possibility this study explores using country as a proxy comparison. China’s state-led AI strategy has actively promoted AI adoption as a national priority, a policy context that has been associated with high levels of public familiarity and optimism toward AI technology (Xu et al., 2025), while Vietnam has experienced a slower and less ideologically driven integration of AI into its media landscape (Triệu, 2025). These contrasting yet culturally related environments make China and Vietnam a well-suited pair for testing whether the cultural-psychological mechanisms proposed in cross-cultural research on technology trust generalize beyond Western contexts and vary in theoretically predictable ways within non-Western societies.

This study focuses on journalism and communication students rather than a broader audience sample because most of these students will go on to become practicing journalists, and their perceptions of generative AI are likely to shape future newsroom practices and professional norms around AI-generated content. The present study addresses this gap by investigating how journalism and communication students in China and Vietnam evaluate the quality of AI-generated news produced by DeepSeek compared to human-written news by professional journalists, under blind conditions. The study aims not merely to document evaluative differences but to examine the cultural-psychological mechanisms through which cultural dimensions modulate perceptions of AI-generated content. The following research questions guide the study:

RQ1: Do journalism and communication students perceive quality differences between AI-generated news and human-written news?

RQ2: Does this difference vary by news category?

RQ3: Are there cross-national differences between Chinese and Vietnamese students in how they evaluate AI-generated news versus human-written news?

## 2 Materials and methods

### 2.1 Research design

This study employs a mixed experimental design to investigate how journalism and communication students in China and Vietnam evaluate the quality of human-written versus AI-generated news texts. The overall design follows a 2 (Country: China/Vietnam) × 2 (Author Type: AI/Human) × 4 (News Type) factorial structure. Country served as a between-subjects variable, while Author Type and News Type were within-subjects variables. Throughout the experiment, participants read all news texts without being informed of the author’s identity and rated their quality accordingly.

### 2.2 Participants

Since one of the core analyses involves testing the interaction effect between country and author type, an a priori power analysis was conducted using G*Power, based on a 2 (Country) × 2 (Author Type) mixed-design repeated-measures ANOVA. With a significance level of α = 0.05, power of 1 − β = 0.95, and effect size *f* = 0.25, selected based on Cohen’s (1988) convention for a medium effect size in the absence of prior effect-size estimates specific to this cross-national comparison, the minimum required sample size was 54 participants.

Chinese participants were recruited through on-campus channels and online platforms at Guangxi University. Vietnamese participants were recruited with the assistance of a Director of the School of Journalism and Communication, University of Social Sciences and Humanities, Vietnam National University, Hanoi, who distributed the questionnaire to journalism and communication students in Vietnam (see Acknowledgments). All procedures, including recruitment and data collection in both countries, were conducted under the ethical approval obtained from Guangxi University, and informed consent was obtained from all participants, including those in Vietnam, prior to data collection. Inclusion criteria were as follows: (1) aged 18 or older, (2) native speakers of the language in which the questionnaire was administered, and (3) currently enrolled in a journalism or communication-related program at a university. Questionnaires were completed by 78 participants in China and 67 participants in Vietnam. Following data screening, four Chinese responses and one Vietnamese response were excluded due to evidence of careless responding (e.g., selecting the same response option across all news quality items), yielding a final sample of 140 valid responses (74 from China, 66 from Vietnam) for analysis. Aside from two participants who selected “uncertain” on the news exposure frequency item (treated as missing for analyses involving that variable), no further missing data or outlier exclusions were necessary.

Participants ranged in age from 18 to 48 years, with 55% identifying as female and 45% as male. The sample consisted of university students enrolled in journalism or communication-related programs, including both undergraduate and postgraduate (master’s-level) students, which accounts for the relatively wide age range observed. The final sample size substantially exceeded the minimum requirement. The study was conducted in accordance with the Declaration of Helsinki and approved by the Institutional Review Board of Guangxi University (protocol code GXU-2025-117; approved on 9 December 2025). Informed consent was obtained from all participants prior to data collection.

### 2.3 Materials

The experimental materials consisted of news texts, which also represent the type of informational content commonly encountered in journalism and communication courses. Materials were developed in three steps. First, authentic news articles written by professional journalists were selected. Second, the core information from each article was extracted and organized using the five Ws framework (Who, What, When, Where, and Why), which was then used to construct prompts for DeepSeek to generate corresponding news texts. Third, questionnaires were prepared in both Chinese and Vietnamese.

DeepSeek was selected as the AI tool for several reasons. First, it is a large language model developed by a Chinese AI company and has demonstrated performance comparable to leading international models across multiple benchmarks (Guo et al., 2025). Second, DeepSeek reflects the actual AI tool environment of the student participants in this study. Chinese university students face both regulatory and accessibility constraints when using generative AI. Restrictions on tools such as ChatGPT have led many students to rely on domestic alternatives (Xie et al., 2025). According to Reuters (2025), several Chinese universities, including Shenzhen University, Zhejiang University, Shanghai Jiao Tong University, and Renmin University of China, have launched DeepSeek-based courses or integrated the tool into coursework and teaching practices since its public release in January 2025, reflecting its growing institutional presence in Chinese higher education. Third, although Vietnamese students have relatively open access to international tools such as ChatGPT, DeepSeek has also gained a specific foothold among Vietnamese AI users. According to a 2025 nationwide survey of Vietnamese consumers, DeepSeek, alongside Gemini, is specifically favored as a study companion for learning new skills, and students report the highest AI adoption rate (92%) of any consumer segment surveyed in Vietnam (Ngo, 2025). This suggests that DeepSeek carries meaningful ecological relevance for the Vietnamese student population specifically, rather than being solely a China-specific tool, supporting the cross-national comparability of the study design.

News samples were drawn from Beijing News^1^ in China and VnExpress^2^ in Vietnam. Both are widely recognized mainstream outlets in their respective countries, selected for their representativeness and editorial quality. Four news categories were included for each country: nature, health, society, and finance. Text length was controlled between 100 and 400 words to ensure comparability across categories.

After extracting the five Ws from each selected article, structured prompts were entered into DeepSeek to generate the AI news texts. Figure 1 provides a screenshot illustrating how prompts were constructed for a finance news item. The prompt provided to DeepSeek was structured as follows. What: upgraded China-EU export control consultations were held. Why: to implement consensus reached by Chinese and EU leaders on trade and economic issues, address concerns in the area of export controls, and promote supply chain stability. When: October 31 to November 1, 2025. Who: Director General Jiang Qianliang of the Ministry of Commerce’s Bureau of Industry and Security, and Deputy Director General Redonnet of the European Commission’s Directorate-General for Trade. Where: Brussels, Belgium. DeepSeek was then instructed to write a news report of approximately 150 words based on the above elements, including a headline. The target length specified in this instruction varied across the 16 article pairs, calibrated in each case to approximate the length of the corresponding human-written original (see Supplementary Table 3 for the complete set of prompts used). Table 1 presents the headlines of both the journalist-written and AI-generated versions across all news categories for both countries.

**FIGURE 1:**
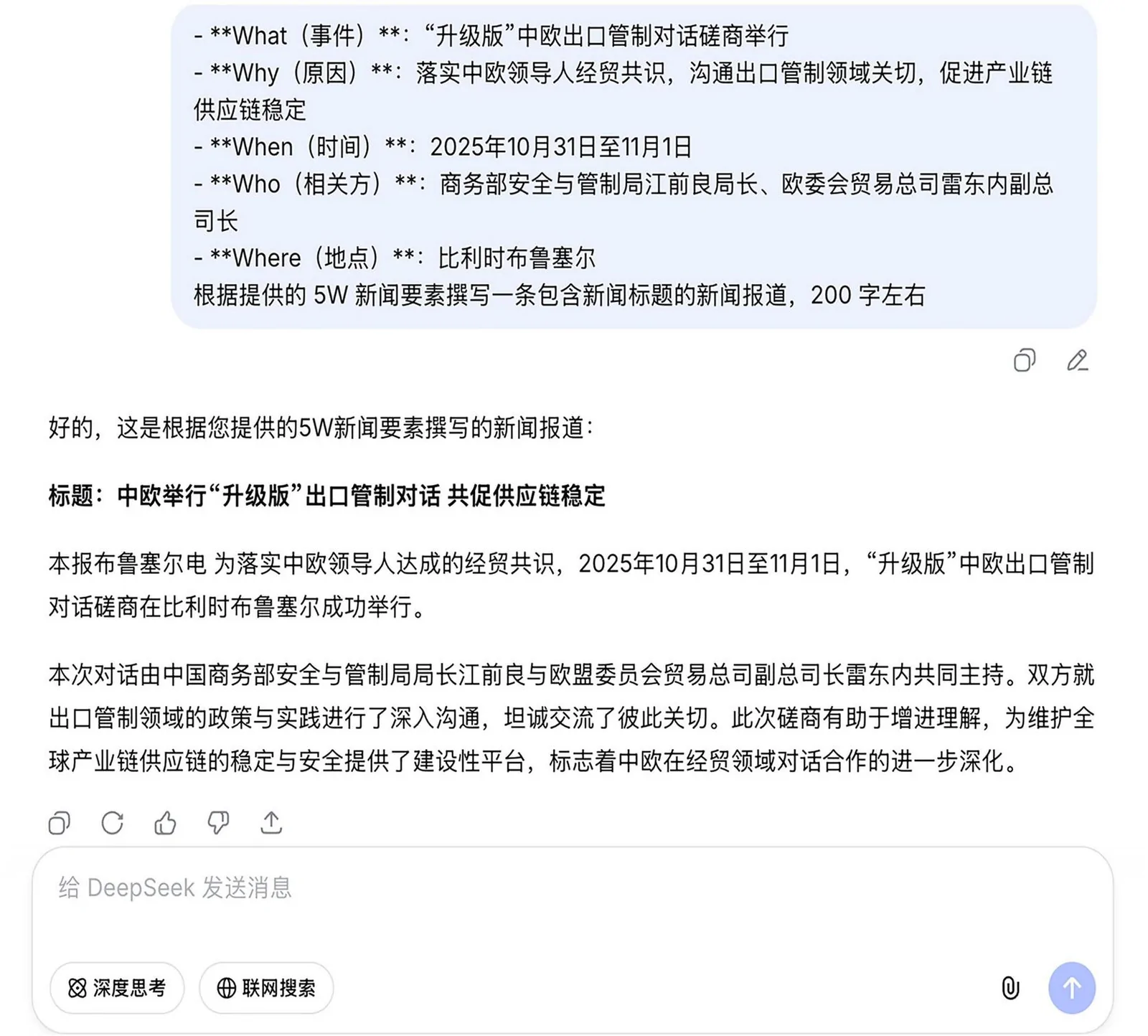
Screenshot: example of prompts posted to DeepSeek.

**TABLE 1:** News headlines of journalist-written and AI-generated articles across topic categories and countries.

Country	Category	Journalist-Written Headline	AI-generated headline
China	Nature	“Supermoon” Hangs Over the Ancient Capital’s Night Sky	The Largest “Supermoon” of 2025 Appears in the Night Sky
	Health	NHSA^1^: 2025 Drug Formulary Negotiations Successfully Concluded	2025 National Medical Insurance Formulary Revision Completed; New List to Take Effect on New Year’s Day
	Society	Couple Hikes for Autumn Scenery; One Gets Lost, Firefighters Launch Emergency Search	Woman Lost While Hiking in Beijing’s Mentougou; Firefighters Complete Successful Nighttime Rescue
	Finance	China-EU Export Control Dialogue Held in Brussels	China and EU Hold “Upgraded” Export Control Dialogue to Jointly Promote Supply Chain Stability
Vietnam	Nature	Rare Hornbill Bird Wanders into Residential Home	Residents and Authorities Cooperate to Successfully Rescue and Release Rare Hornbill Back to Forest
	Health	Substandard Painkiller and Fever Reducer Batch Recalled	Drug Administration Urgently Orders Nationwide Recall of Children’s Medicine Padobaby
	Society	Truck Overturns on Nha Trang–Cam Ranh Road	Truck Overturns on Cu Hin Mountain Road, Causing Prolonged Traffic Congestion
	Finance	Vietnam Welcomes US Decision Not to Adjust Trade Policy	US Does Not Apply Trade Adjustment Measures; Vietnam Welcomes the Decision

NHSA^1^, National Healthcare Security Administration. Headlines translated from Chinese and Vietnamese by the authors.

To assess objective differences between the resulting human-written and AI-generated stimulus texts, all 16 articles (2 countries × 4 topics × 2 authorship conditions) were analyzed using natural language processing tools. Chinese text was segmented using jieba (version 0.42.1), and Vietnamese text was segmented using underthesea (version 9.5.0). Because Vietnamese is written with syllable-level spacing (e.g., “Việt Nam” and “thúóng mại” are each single lexical words composed of two space-separated syllables), word counts for Vietnamese texts reflect linguistically segmented words rather than space-delimited syllables, and are therefore not directly comparable to a simple syllable count.

For each article, we computed headline length, article length, number of sentences, average sentence length, lexical diversity [type-token ratio (TTR) and the Measure of Textual Lexical Diversity (MTLD); lexicalrichness package, version 0.5.1], and the frequency of explicit source-attribution markers (e.g., “according to,” “officials said”). To assess whether DeepSeek may have reproduced rather than generated the corresponding source article, lexical (Jaccard) overlap between each human-written and AI-generated pair was also computed.

As summarized in Table 2 (full topic-level results in Supplementary Table 1), AI-generated headlines were consistently longer than human-written headlines in both countries (China: 22.3 vs. 18.0 characters; Vietnam: 10.0 vs. 7.5 words). Article length showed an inconsistent pattern across the two countries: AI-generated articles were longer than human-written articles in China (196.5 vs. 149.0 characters) but shorter in Vietnam (171.3 vs. 200.5 words), indicating that this feature reflected the specific stimulus materials rather than a general property of AI-generated text. AI-generated articles showed higher lexical diversity (MTLD) than human-written articles in both China (118.4 vs. 78.2) and Vietnam (183.3 vs. 132.9), a pattern that runs counter to the assumption that AI-generated text is inherently more homogeneous or repetitive than human-written text. Human-written articles contained more explicit source-attribution markers than AI-generated articles in both countries (China: 1.50 vs. 0.25; Vietnam: 2.25 vs. 1.25). Jaccard overlap between each human-AI article pair ranged from 0.25 to 0.43 (Supplementary Table 2), indicating that the AI-generated articles were not verbatim reproductions of the source articles. This lexical overlap measure speaks only to wording similarity, however, and does not establish equivalence in factual content between the human-written and AI-generated versions.

**TABLE 2:** Summary of objective textual features of human-written and AI-generated articles, by country (averaged across the four topic categories).

Country	Authorship	Headline length^a^	Length^b^	TTR	MTLD	Source markers^c^
China	Human-Written	18.0 characters	149.0 characters	0.76	78.2	1.50
China	AI-Generated	22.3 characters	196.5 characters	0.79	118.4	0.25
Vietnam	Human-Written	7.5 words	200.5 words	0.60	132.9	2.25
Vietnam	AI-Generated	10.0 words	171.3 words	0.67	183.3	1.25

^a^Headline length is reported in characters for Chinese articles and words for Vietnamese articles, reflecting the two writing systems.

^b^Article body length, reported in the same units as headline length. TTR = type-token ratio; MTLD = Measure of Textual Lexical Diversity.

^c^Count of explicit source-attribution markers (e.g., “according to,” “officials said”) per article. Values are averaged across the four topic categories (nature, health, society, finance); the full breakdown by topic is reported in Supplementary Table 1.

### 2.4 Procedure

The experiment proceeded in three stages. First, participants completed a brief survey on their news consumption habits, including news exposure frequency, interest, and trust; the news trust item (5-point scale) was reverse-scored (6 - raw score) so that higher values indicated greater trust in news media. Second, they read news articles across four categories in sequence. Within each category, participants read one human-written article and one AI-generated article, then rated the quality of both. Finally, participants provided demographic information.

To ensure objective evaluations, the author’s identity was not disclosed to participants. They were only told that the study examined college students’ assessments of online news quality. All news texts were presented in a uniform format (Figure 2), and bylines were removed to prevent source information from influencing judgments, as prior research has shown that author identity can significantly affect how audiences assess news credibility (Wang and Huang, 2024). The order in which human-written and AI-generated articles were presented was not randomized on a per-participant basis; instead, presentation order alternated across the four topic categories (e.g., human-written article presented first for Nature, AI-generated article presented first for Health, and so on), such that each authorship condition appeared first in two of the four categories. Within each news category, articles were accompanied by images sourced directly from the original reports in Beijing News and VnExpress; for two Chinese articles for which the original report did not include an image, a topic-appropriate substitute image was used instead. Due to third-party copyright considerations, the full text of the original news articles used as experimental stimuli is not included in Supplementary material.

**FIGURE 2:**
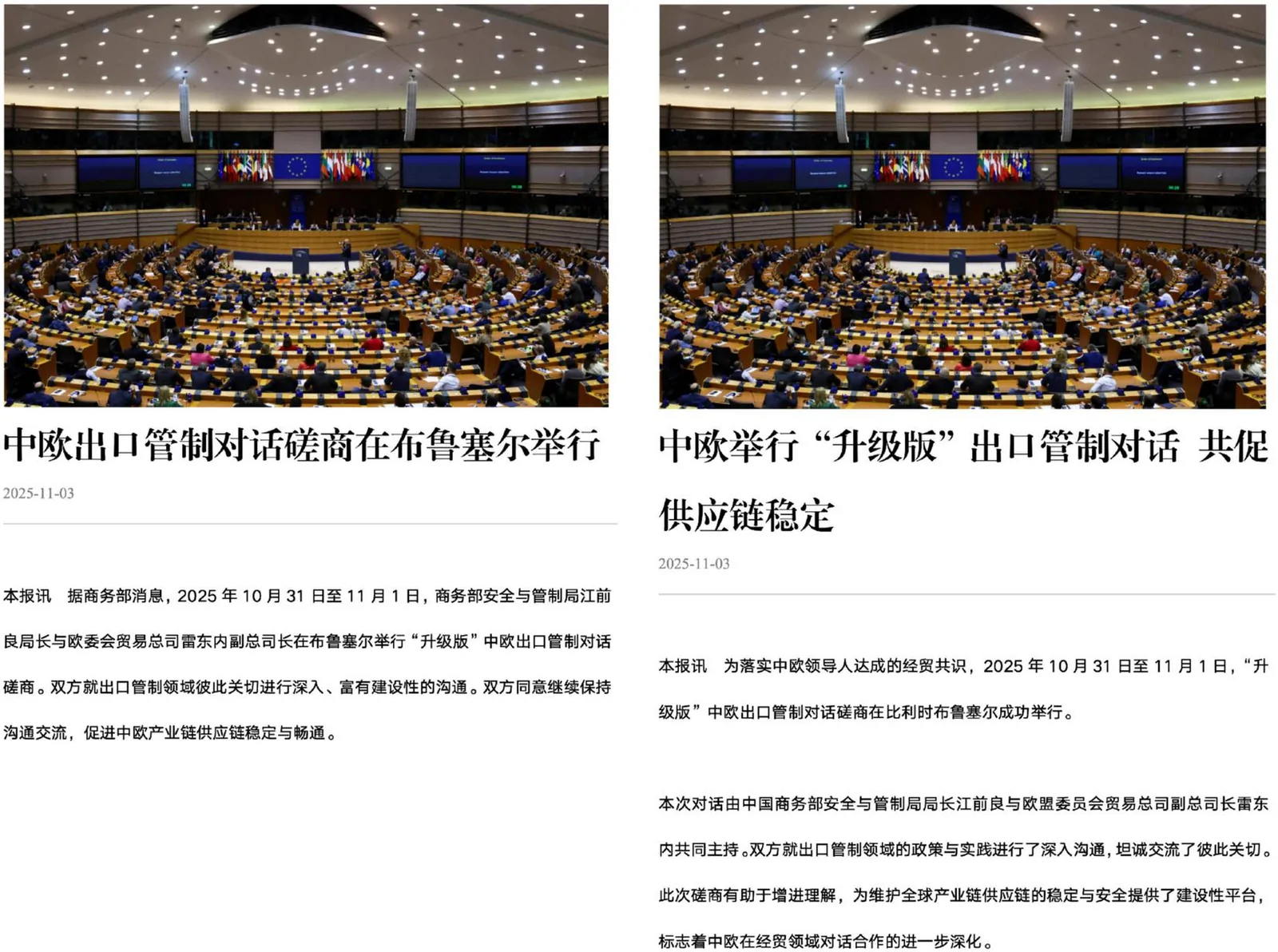
Examples of news article templates used in the study. Human-written news (left) and AI-generated news (right). Source of the human-written article: Beijing News. The accompanying image is sourced from the European Parliament Multimedia Centre (© European Union – 2025).

### 2.5 Measures

News text quality was assessed using a seven-item scale adapted from Baptista et al. (2025), who developed their quality indicator based on the framework of Arapakis et al. (2016), which conceptualizes news quality across three dimensions: readability, informativeness, and style. Item content was translated into Chinese and Vietnamese without substantive modification to wording or scope. The complete set of items in English, Chinese, and Vietnamese is provided in Supplementary Data Sheet 1.

The Vietnamese version of the questionnaire was prepared via machine translation (Google Translate), followed by back-translation into English to confirm semantic equivalence with the original items; no meaningful discrepancies were identified. As an additional safeguard, the Vietnamese version was reviewed by native-speaking Vietnamese students who were not involved in the main study, and minor revisions were made to improve the naturalness of phrasing.

The scale comprised seven items rated on a 7-point Likert scale (1 = Strongly Disagree, 7 = Strongly Agree): (1) ease of reading and comprehension, (2) consistent and orderly presentation of facts, (3) headlines that accurately reflect the content, (4) sufficient and comprehensive information, (5) balanced use of sources, (6) language complexity and difficulty (reverse-scored), and (7) clarity of punctuation and sentence structure.

Overall quality scores were computed as the unweighted mean of the seven items, with the linguistic expression item reverse-scored prior to aggregation (transformed as 8—raw score), such that higher values consistently indicated more favorable evaluations across all items.

Internal consistency was acceptable for both the human-written (Cronbach’s α = 0.79) and AI-generated (α = 0.78) news scales overall, and was broadly comparable across the Chinese (human α = 0.85; AI α = 0.86) and Vietnamese (human α = 0.77; AI α = 0.76) versions of the questionnaire. Exploratory factor analysis (KMO = 0.88–0.91; Bartlett’s test *p*s < 0.001) revealed a consistent two-factor structure for both news types: six items (readability, factual accuracy, headline relevance, coverage adequacy, source usage, and punctuation) loaded strongly onto a single content/structure quality factor (loadings = 0.76–0.94), while the linguistic expression item loaded exclusively and strongly (loadings = 0.96–0.97) onto a distinct second factor. This pattern suggests that perceived linguistic simplicity constitutes a conceptually distinct dimension rather than a measurement deficiency; the seven-item unweighted mean was therefore retained as the overall quality score.

### 2.6 Data analysis

Data analysis was conducted using SPSS 29.0.2. The primary analysis employed a 2 (Country: China/Vietnam, between-subjects) × 2 (Authorship: AI/Human, within-subjects) × 4 (Topic: Nature/Health/Society/Finance, within-subjects) mixed-design ANOVA on overall news quality scores. This approach allowed the Country × Authorship, Country × Topic, Authorship × Topic, and Country × Authorship × Topic interactions to be tested directly within a single model. Mauchly’s test indicated that the assumption of sphericity was violated for the Topic and Topic × Authorship effects (both *p* < 0.001); Greenhouse-Geisser corrected degrees of freedom are therefore reported for these effects. Levene’s test indicated homogeneity of variance across all cells (all *p* > 0.05). Shapiro-Wilk tests indicated significant deviations from normality in 9 of the 16 Country × Topic × Authorship cells (*p*s < 0.05); however, skewness (range: −1.20 to −0.24) and kurtosis (range: −0.71 to 2.15) remained within generally accepted bounds for parametric analysis, and mixed-design ANOVA is considered robust to non-normality of this magnitude given the sample sizes employed here (Blanca et al., 2017). Significant interactions were followed up with pairwise comparisons using estimated marginal means, with Bonferroni correction for multiple comparisons. Effect sizes are reported as partial eta squared (partial η^2^) throughout. To directly test whether individual-level factors interacted with authorship, gender, age, grade level, news exposure frequency, news interest, and news trust were entered simultaneously as predictors in three linear regression models predicting human-written news quality, AI-generated news quality, and the AI-minus-human difference score, respectively. The difference-score model provides a direct test of each predictor’s interaction with Authorship.

## 3 Results

### 3.1 Mixed-design ANOVA results

A 2 (Country) × 2 (Authorship) × 4 (Topic) mixed-design ANOVA was conducted on overall news quality scores. All effects are summarized in Table 3, and descriptive statistics for all Country × Topic × Authorship combinations are reported in Table 4.

**TABLE 3:** Mixed-design ANOVA results for overall news quality.

Source	df	*F*	*p*	Partial η ^2^	90% CI^b^
Country	1, 138	6.41	0.013	0.044	[0.005, 0.111]
Authorship	1, 138	19.88	<0.001	0.126	[0.051, 0.213]
Topic ^a^	2.68, 369.96	4.60	0.005	0.032	[0.006, 0.062]
Country × Authorship	1, 138	7.18	0.008	0.049	[0.007, 0.119]
Country × Topic ^a^	2.68, 369.96	4.22	0.008	0.030	[0.005, 0.058]
Authorship × Topic ^a^	2.66, 367.29	11.83	<0.001	0.079	[0.036, 0.121]
Country × Authorship × Topic ^a^	2.66, 367.29	1.87	0.141	0.013	[0.000, 0.033]

^a^Greenhouse-Geisser corrected degrees of freedom are reported because Mauchly’s test indicated a violation of sphericity for effects involving Topic (all *p* < 0.001).

^b^90% confidence intervals for partial η^2^, computed from the noncentral F distribution, following Steiger (2004).

**TABLE 4:** Descriptive statistics and 95% confidence intervals for perceived news quality by country, topic, and authorship.

Topic	Authorship	China M ± SD [95% CI]	Vietnam M ± SD [95% CI]
Nature	Human-Written	4.48 ± 0.83 [4.28, 4.67]	5.13 ± 0.93 [4.90, 5.36]
Nature	AI-Generated	5.14 ± 0.79 [4.96, 5.33]	5.44 ± 0.79 [5.25, 5.63]
Health	Human-Written	5.10 ± 0.85 [4.90, 5.30]	5.32 ± 0.94 [5.09, 5.55]
Health	AI-Generated	5.04 ± 0.86 [4.84, 5.24]	5.29 ± 0.90 [5.07, 5.51]
Society	Human-Written	4.88 ± 0.93 [4.67, 5.10]	5.24 ± 0.89 [5.02, 5.45]
Society	AI-Generated	5.33 ± 0.92 [5.11, 5.54]	5.40 ± 0.78 [5.21, 5.59]
Finance	Human-Written	4.93 ± 0.78 [4.75, 5.12]	5.29 ± 0.71 [5.12, 5.47]
Finance	AI-Generated	5.22 ± 0.81 [5.03, 5.41]	5.19 ± 0.82 [4.99, 5.39]

China *n* = 74; Vietnam *n* = 66. Values in brackets are 95% confidence intervals for each cell mean.

The three-way Country × Authorship × Topic interaction was not significant, *F*(2.66, 367.29) = 1.87, *p* = 0.141, partial η^2^ = 0.013, indicating that the pattern of topic-specific authorship differences did not differ substantially between the two countries.

The Authorship × Topic interaction was significant, *F*(2.66, 367.29) = 11.83, *p* < 0.001, partial η^2^ = 0.079 (Figure 3). AI-generated news received higher ratings than human-written news in the Nature (ΔM = 0.49) and Society (ΔM = 0.30) categories, a smaller advantage in Finance (ΔM = 0.09), and no advantage in Health (ΔM = −0.05), full simple-effect estimates with 95% confidence intervals are reported in Table 5.

**FIGURE 3:**
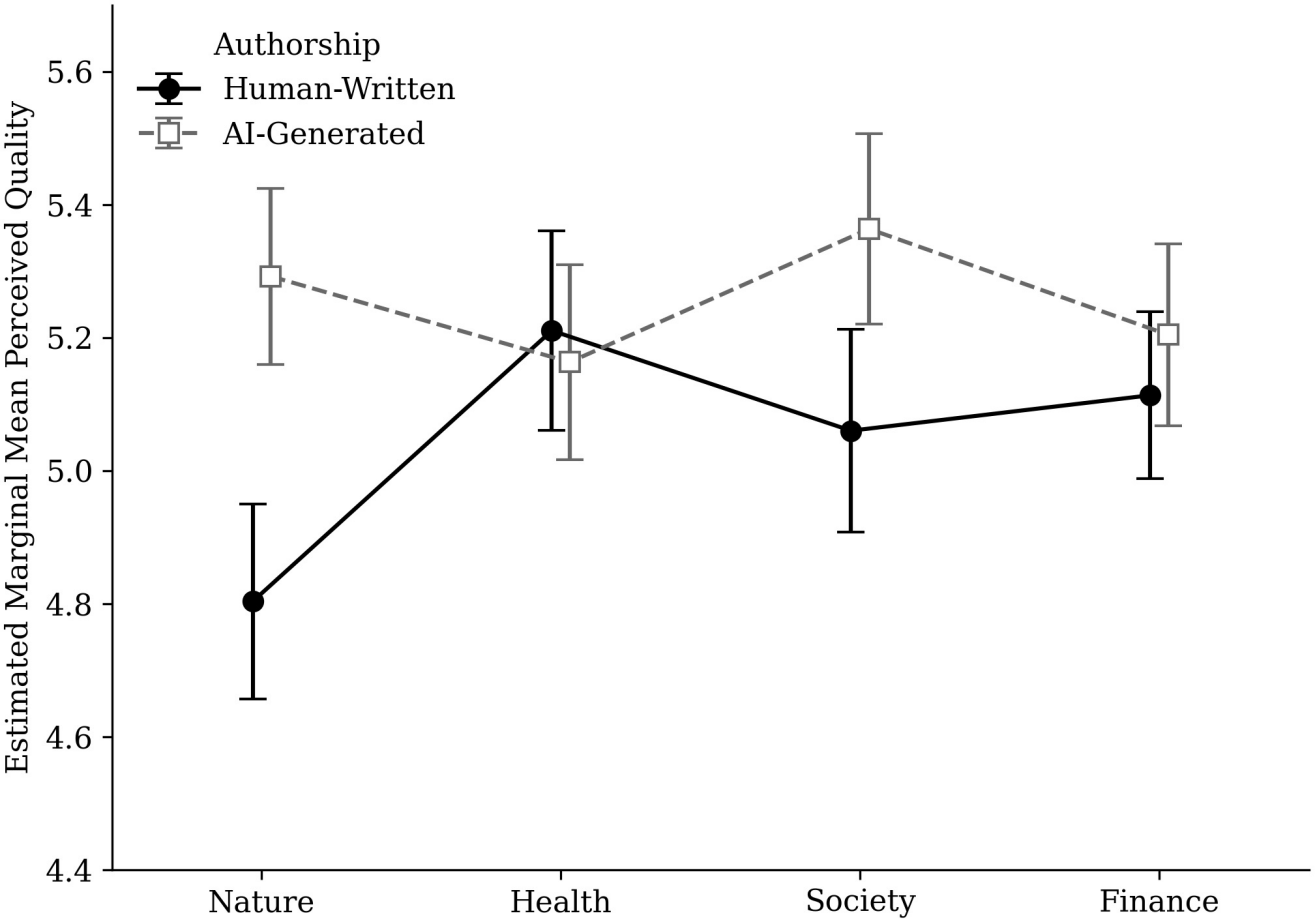
Estimated marginal mean perceived quality of AI-generated and human-written news by topic (error bars represent 95% confidence intervals).

**TABLE 5:** Direct estimates of the authorship (AI-Human) simple effect within each country and each topic.

Simple effect	n	Mean Difference (AI-human)	95% CI	*t*	*p*
Country: China	74	0.334	[0.230, 0.439]	6.37	<0.001
Country: Vietnam	66	0.083	[−0.076, 0.243]	1.04	0.301
Topic: Nature	140	0.499	[0.350, 0.648]	6.61	<0.001
Topic: Health	140	−0.048	[−0.193, 0.097]	−0.65	0.515
Topic: Society	140	0.311	[0.143, 0.480]	3.65	<0.001
Topic: Finance	140	0.102	[−0.036, 0.240]	1.46	0.147

Positive values indicate higher perceived quality for AI-generated news relative to human-written news. Country-level estimates are averaged across the four topics; topic-level estimates are averaged across both countries. These simple-effect estimates directly quantify the Country × Authorship and Authorship × Topic interactions reported in Table 3.

The Country × Authorship interaction was also significant, *F*(1, 138) = 7.18, *p* = 0.008, partial η^2^ = 0.049 (Figure 4). Chinese participants showed a substantially larger AI-preference (human *M* = 4.85, AI *M* = 5.18, ΔM = 0.33, 95% CI [0.23, 0.44]) than Vietnamese participants (human *M* = 5.25, AI *M* = 5.33, ΔM = 0.08, 95% CI [−0.08, 0.24] (n.s.); these statistics are reported in Table 5).

**FIGURE 4:**
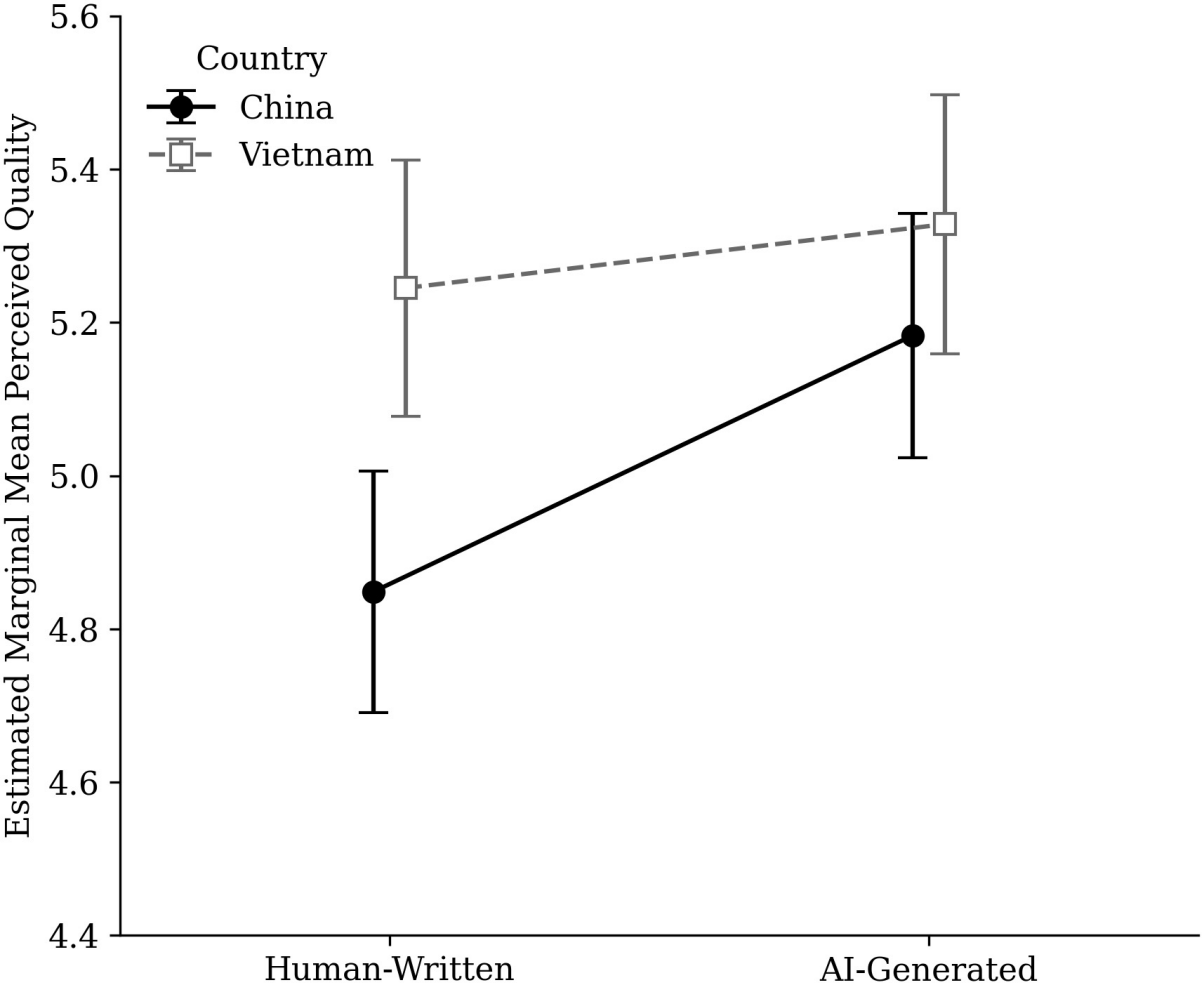
Estimated marginal mean perceived quality of AI-generated and human-written news by country (error bars represent 95% confidence intervals).

The Country × Topic interaction was significant, *F*(2.68, 369.96) = 4.22, *p* = 0.008, partial η^2^ = 0.030. As shown in Table 4, the cross-national gap in perceived quality was largest for Nature news (China *M* = 4.81, Vietnam *M* = 5.28, ΔM = 0.47) and comparatively smaller for Health, Society, and Finance news (ΔMs = 0.16–0.24).

Main effects were also significant: Authorship, *F*(1, 138) = 19.88, *p* < 0.001, partial η^2^ = 0.126, with AI-generated news (*M* = 5.26) rated higher than human-written news (*M* = 5.05) overall; Country, *F*(1, 138) = 6.41, *p* = 0.013, partial η^2^ = 0.044, with Vietnamese participants (*M* = 5.29) rating news higher overall than Chinese participants (*M* = 5.02); and Topic, *F*(2.68, 369.96) = 4.60, *p* = 0.005, partial η^2^ = 0.032.

### 3.2 Comparison across quality indicators

Table 6 reports the seven quality indicators. AI-generated news scored significantly higher than human-written news on perceived readability [*t*(139) = 2.05, *p* = 0.042, d*^z^* = 0.17], perceived headline relevance [*t*(139) = 7.51, *p* < 0.001, d*^z^* = 0.64], perceived coverage adequacy [*t*(139) = 4.40, *p* < 0.001, d*^z^* = 0.37], and perceived punctuation and sentence structure [*t*(139) = 3.71, *p* < 0.001, d*^z^* = 0.31]. Among these, perceived headline relevance showed the largest effect size, followed by perceived coverage adequacy and perceived punctuation and sentence structure. No significant differences were found on perceived factual accuracy (*p* = 0.126), perceived source usage (*p* = 0.569), or perceived linguistic simplicity (*p* = 0.121).

**TABLE 6:** Perceived quality of AI-generated and human-written news across seven quality indicators.

Indicator	Human M ± SD	AI M ± SD	Mean diff. (AI-human)	95% CI	*t*	*p*	d*^z^*
Perceived readability	5.43 ± 1.00	5.55 ± 0.99	0.12	[0.00, 0.24]	2.05	0.042	0.17
Perceived factual accuracy	5.38 ± 0.93	5.48 ± 0.93	0.10	[−0.03, 0.23]	1.54	0.126	0.13
Perceived headline relevance	5.00 ± 1.08	5.60 ± 0.96	0.60	[0.44, 0.76]	7.51	<0.001	0.64
Perceived coverage adequacy	5.15 ± 1.03	5.47 ± 0.92	0.31	[0.17, 0.46]	4.40	<0.001	0.37
Perceived source usage	5.15 ± 0.95	5.19 ± 1.02	0.04	[−0.10, 0.18]	0.57	0.569	0.05
Perceived linguistic simplicity	3.90 ± 1.40	4.01 ± 1.49	0.11	[−0.03, 0.26]	1.56	0.121	0.13
Perceived punctuation and sentence structure	5.24 ± 1.04	5.46 ± 0.97	0.22	[0.10, 0.34]	3.71	<0.001	0.31

d*^z^* = Cohen’s *d* for repeated-measures designs, computed as the mean paired difference divided by the standard deviation of the paired differences. Labels reflect participants’ subjective ratings of perceived quality and do not imply independent verification of objective textual properties such as actual factual accuracy or source reliability.

### 3.3 Effects of individual-level factors

To examine whether individual-level factors moderated the effect of authorship on perceived news quality, gender, age, grade level, news exposure frequency, news interest, and news trust were entered simultaneously as predictors in three linear regression models: one predicting human-written news quality, one predicting AI-generated news quality, and one predicting the AI-minus-human difference score. The difference-score model provides a direct test of whether each predictor’s effect differs between the two authorship conditions (Table 6).

Gender significantly predicted both human-written (*B* = −0.29, *p* = 0.011) and AI-generated (*B* = −0.45, *p* < 0.001) news quality, with male participants giving lower ratings overall. However, gender did not significantly predict the AI-minus-human difference score (*B* = −0.16, *p* = 0.128), indicating that the effect of gender did not differ significantly between the two authorship conditions.

News interest significantly predicted both human-written (*B* = 0.36, *p* < 0.001) and AI-generated (*B* = 0.23, *p* < 0.001) news quality, but the coefficient was substantially larger for human-written news; this asymmetry was confirmed directly, with news interest significantly predicting the difference score (*B* = −0.14, *p* = 0.021).

News trust followed a similar pattern: it significantly predicted human-written news quality (*B* = 0.17, *p* = 0.002) but not AI-generated news quality (*B* = 0.06, *p* = 0.352). This asymmetry was likewise confirmed directly, with news trust significantly predicting the difference score (*B* = −0.12, *p* = 0.021).

Age, grade level, and news exposure frequency did not significantly predict human-written news quality, AI-generated news quality, or the difference score (all *p* > 0.07; Table 6).

## 4 Discussion

This study examines how journalism and communication students in China and Vietnam, two culturally distinct yet geographically proximate societies, evaluate the quality of AI-generated news and human-written news across multiple topic categories and quality dimensions. The findings reveal several theoretically meaningful patterns that extend and enrich existing research on perceptions of AI-generated news, particularly by introducing cross-national evidence from non-Western cultural contexts.

### 4.1 Overall preference for AI-generated news and cross-national divergence

In general, participants gave a higher evaluation of AI-generated news than human-written news. This result is consistent with the previous study findings based on samples from the Iberian Peninsula (Baptista et al., 2025). However, this pattern was not uniform across countries: a significant Country × Authorship interaction (*F* = 7.18, *p* = 0.008) indicated that Chinese participants showed a substantially larger preference for AI-generated news (delta *M* = 0.33, 95% CI [0.23, 0.44]) than Vietnamese participants, whose evaluations of the two content types did not differ significantly (delta *M* = 0.08, 95% CI [−0.08, 0.24]). This suggests that the tendency to rate AI-generated news more favorably is not universal, and may plausibly be shaped by the media environment, technological familiarity, and cultural background of students.

It is worth noting that China’s research results coincide with broader regional trends. Maslej et al. (2025) reported that 83% of Chinese respondents believe that the benefits of AI products and services outweigh the disadvantages, which is much lower in Western countries. This high degree of acceptance is consistent with a broader cultural orientation that scholars describe as techno-optimism. Cross-cultural research has similarly found that collectivist societies tend to exhibit greater AI optimism than individualist Western societies, partly due to cultural emphases on societal harmony and long-term national planning (Brauner et al., 2025). This trend appears particularly prominent in China, where state-led technological development and narratives of national modernization may have contributed to strong public enthusiasm for AI as a symbol of national progress (Xu et al., 2025). Unlike in many Western countries, where public discourse around AI tends to foreground risks, ethical concerns, and job displacement, China’s state-led technology strategy has actively promoted AI adoption as integral to national rejuvenation, a context that may frame embracing AI tools as both patriotic and progressive.

From a cultural psychological perspective, this pattern is potentially consistent with the lens of interdependent self-construal (Markus and Kitayama, 1991). In collectivist cultures, the self is defined in relation to significant others and collective goals, and individuals are motivated to align their attitudes and behaviors with perceived social expectations. To the extent that the Chinese state frames AI as a cornerstone of national rejuvenation, individuals with an interdependent self-construal may internalize AI acceptance not merely as a personal preference but as an expression of social identity and civic belonging. This suggests that Chinese students’ higher ratings of AI-generated news may reflect not only a genuine perception of quality but also a culturally embedded disposition to endorse content associated with nationally sanctioned technological progress; however, self-construal was not directly measured among individual participants in this study, and this interpretation remains speculative. China’s markedly higher national-level long-term orientation score (LTO = 87) compared to Vietnam (LTO = 57) (Hofstede et al., 2010) offers one possible explanation: individuals in high-LTO cultures tend to evaluate present-day technologies through the lens of future societal potential, fostering greater tolerance for current limitations and a more optimistic appraisal of AI-generated content as part of an emerging social infrastructure.

Although Vietnam and China have some values in common, such as being influenced by Confucianism, Vietnam’s integration of AI into media and education has proceeded more slowly and with less ideological emphasis (Triệu, 2025). This contextual difference may help account for why Vietnamese respondents did not show a similar preference for AI-generated content, although other unmeasured country-level factors, such as differences in journalism curricula, general AI literacy, or prior experience with DeepSeek specifically, could also plausibly contribute to this divergence and cannot be ruled out with the present data.

### 4.2 Topic-specific patterns in AI news evaluation

Variations in the performance of AI-generated news across topic categories may be explained through two complementary mechanisms operating at the textual and cultural levels, respectively. At the textual level, research indicates that AI-generated text prioritizes information delivery over emotional engagement and features more consistent syntactic structures, granting it structural clarity advantages in event-based narrative texts (Terčon and Dobrovoljc, 2025). This is broadly consistent with the present textual analysis: in the Chinese stimulus materials, for example, the AI-generated health article divided the same information into considerably shorter, more numerous sentences than its human-written counterpart (see Materials), a pattern that may enhance perceived readability. Nature and society news are characterized by linear, event-driven narratives that align closely with the standardized textual patterns learned by large language models, potentially enabling AI to leverage its structural strengths in syntactic regularity and paragraph organization. In contrast, no significant quality differences between AI-generated and human-written news were observed in health and finance news, which may reflect similarities in professional norms and linguistic registers across these genres. Health and finance reporting features objective, formal, and information-dense language, which aligns with the default output tendencies commonly reported for large language models (Sardinha, 2024). Studies show that compared with the more engaging and interactive writing of humans, AI-generated text matches informational and academic registers with higher lexical density, nominalization, and explicit cohesion devices (Georgiou, 2025). To the extent that target genres require these same formal and objective linguistic features, the stylistic gap between AI-generated and human-written text may narrow, potentially reducing perceived quality differences.

At the cultural level, a further mechanism specific to East Asian contexts may help explain why AI’s advantages did not extend to health and finance news. Research on heuristic information processing suggests that individuals routinely rely on an authority heuristic when evaluating content credibility, automatically linking perceived trustworthiness to the official or institutional standing of the source (Sundar, 2008; Metzger and Flanagin, 2013). In high power distance cultures, this authority heuristic may be particularly pronounced: individuals may develop a culturally reinforced tendency to assess information credibility through the lens of source hierarchy, discounting content that cannot be traced to a recognizable institutional authority (Hofstede et al., 2010). China and Vietnam score considerably higher on Hofstede’s Power Distance Index (China: 80; Vietnam: 70) compared to Portugal (63) and Spain (57); however, power distance was not directly measured among individual participants in this study, and its role here remains a plausible but untested interpretation. Health and finance reporting in China and Vietnam is closely tied to state institutions, official data sources, and policy communication frameworks, which may create strong culturally embedded expectations around source authority. Consistent with this account, the present textual analysis found that AI-generated articles contained fewer explicit source-attribution markers than their human-written counterparts in both countries (see Materials); AI-generated news, which frequently relies on vague or absent source citations (Sholpan et al., 2025), may fail to activate the authority heuristic in a positive direction, since it provides no recognizable institutional anchor, potentially triggering a reduction in perceived content credibility instead. This mechanism may offset any structural advantages AI-generated news might otherwise hold in these topic categories. Together, these two mechanisms offer a plausible dual explanation for why AI’s advantage disappears specifically in the health and finance categories: stylistic convergence may narrow the structural gap, while the relative absence of authoritative source attribution may prevent AI from gaining a content-level edge.

### 4.3 Structural advantages and content-level limitations

Further analysis of quality dimensions reveals that AI-generated news outperforms human-written news specifically on dimensions related to textual structure, including perceived headline relevance (d*^z^* = 0.64), coverage adequacy (d*^z^* = 0.37), and punctuation and sentence structure (d*^z^* = 0.31; see Table 6). This pattern is broadly consistent with prior work suggesting that standardized syntactic patterns, well-organized paragraph structures (Herbold et al., 2023), and structurally consistent headlines (Gherhes et al., 2024) in AI-generated text may contribute to higher evaluation scores. Audiences recognize the structural form of AI-generated articles and affirm their organizational clarity (Sholpan et al., 2025). However, AI-generated news shows no corresponding advantages in content-related dimensions: perceived factual accuracy and perceived source usage did not differ significantly between AI-generated and human-written news (Table 6). This is consistent with prior observations that AI-generated news can omit citations from authoritative sources, rely on vague or missing references, and be prone to factual inaccuracies or hallucinatory content (Sholpan et al., 2025; Wang and Huang, 2024).

This structural-content asymmetry diverges from the findings of Baptista et al. (2025), whose study of journalism students in Portugal and Spain found that AI-generated news received higher ratings across nearly all quality dimensions, including content-related ones. This divergence may reflect systematic differences in how readers from high versus low power distance cultures apply source-based heuristics when evaluating news content. In high power distance cultures such as China and Vietnam, individuals tend to show deference to authority, which shapes their decision-making processes (Daniels and Greguras, 2014). When evaluating news content, this cultural disposition may activate a strong authority heuristic: readers automatically link content credibility to the institutional standing of the source, such that even structurally well-formed content lacking traceable authoritative attribution is discounted in quality judgments (Sundar, 2008; Metzger and Flanagin, 2013). In lower power distance contexts such as Portugal and Spain, source hierarchy carries comparatively less evaluative weight, and textual quality itself, including structural clarity and readability, may serve as a more primary basis for quality judgments. This cultural difference in the weight assigned to source authority versus textual quality offers a theoretically grounded explanation for why AI’s content-level limitations were more consequential for East Asian evaluators than for their Southern European counterparts. Beyond cultural factors, methodological differences between studies, including the AI tools used, news topics covered, and participant characteristics, may also contribute to the divergent pattern, suggesting that the generalizability of AI news quality evaluations across cultural and methodological contexts warrants further investigation. Together, these findings suggest that the relationship between AI news quality and cultural evaluation norms represents a theoretically productive area for cross-cultural psychology, warranting systematic investigation across a broader range of cultural contexts.

### 4.4 Cross-national consistency in AI news evaluations

Cross-cultural analysis further shows that there were obvious differences between Chinese and Vietnamese students’ evaluation of human-written news, but their evaluation of AI-generated news was comparatively convergent. It should be noted that this evaluative convergence should not be conflated with convergence in the underlying cultural attitudes, textual properties, or narrative conventions themselves, which were not directly measured in this study; nonetheless, this pattern offers a valuable empirical basis for developing theoretically grounded hypotheses about the cultural and textual mechanisms that may underlie it. This pattern of convergence may be explained from two angles. First, human-written news is deeply influenced by national culture, language habits and news traditions, and these factors vary greatly from country to country (Hanusch and Hanitzsch, 2017; Rafiee et al., 2021), meaning that readers who grow up in different news cultures may apply different criteria when evaluating human-written texts. Second, some research reports that large language models exhibit greater stylistic uniformity across users and contexts than human writers, a pattern observed in creative writing (Anderson et al., 2024), college papers (Moon et al., 2025), and cross-cultural writing tasks (Agarwal et al., 2025) and some scholars have argued that this standardized generation process may reduce the cultural and linguistic variation that distinguishes human writing across nations (Daryani et al., 2026; Lakshmi, 2025). The present textual analysis, however, offers a more complicated picture: AI-generated articles showed higher, not lower, lexical diversity (MTLD) than human-written articles in both China and Vietnam (see Materials), which is inconsistent with a straightforward homogenization account based on reduced lexical variety. It remains possible that AI-generated text converges with itself across cultural contexts along dimensions other than lexical diversity, for instance, narrative structure, framing conventions, or rhetorical style, but this possibility was not directly tested here and would require dedicated cross-linguistic comparison of AI outputs to evaluate.

This cross-national convergence raises a theoretically significant question for cultural psychology. For readers with an interdependent self-construal, which is characteristic of both East Asian (e.g., Chinese) and Southeast Asian (e.g., Vietnamese) cultural contexts (Lim and MaKellams, 2025), the absence of individual authorship in AI-generated text may produce ambivalent psychological responses. On the one hand, the removal of a culturally situated human author may induce a form of cultural estrangement, as the text lacks the relational and contextual anchoring that interdependent readers typically draw upon when evaluating communicative intent and moral standing. On the other hand, precisely because AI-generated text carries no individual voice, regional bias, or personal agenda, it may paradoxically be perceived as more impartial and socially neutral, a form of information that sidesteps the interpersonal and hierarchical dynamics that typically mediate trust judgments in high-context, collectivist cultures. Whether AI-generated news ultimately feels alienating or reassuring to interdependent readers remains an open empirical question, but one with significant implications for understanding how cultural self-construal shapes media reception in the age of generative AI.

Regardless of the specific underlying mechanism, this finding carries broader theoretical implications for cross-cultural psychology: as AI-generated content becomes increasingly prevalent across diverse cultural contexts, it may contribute to a gradual convergence in how audiences from different cultural backgrounds perceive and evaluate media content, potentially reshaping culturally distinct patterns of media reception.

### 4.5 Individual-level factors

At the individual level, gender significantly predicted evaluations of both human-written (*B* = −0.29, *p* = 0.011) and AI-generated (*B* = −0.45, *p* < 0.001) news, with male participants giving lower ratings overall; a direct interaction test, however, indicated that this effect did not differ significantly between the two authorship conditions (*B* = −0.16, *p* = 0.128), suggesting a general rather than authorship-specific effect of gender, broadly consistent with prior research on gender differences in media consumption (Wang and Huang, 2024). A different pattern emerged for news interest and news trust: both factors significantly predicted evaluations of human-written news (interest: *B* = 0.36, *p* < 0.001; trust: *B* = 0.17, *p* = 0.002), but showed a significantly weaker effect (interest: *B* = 0.23, *p* < 0.001) or no significant effect (trust: *B* = 0.06, *p* = 0.352) on evaluations of AI-generated news; direct interaction tests confirmed that both of the interaction effects were statistically reliable (interest: *B* = −0.14, *p* = 0.021; trust: *B* = −0.12, *p* = 0.021; see Table 7).

**TABLE 7:** Regression coefficients predicting human-written news quality, AI-generated news quality, and the AI-minus-human difference score from individual-level factors.

Predictor	B (Human)	95% CI	B (AI)	95% CI	B (Diff)	95% CI
Gender	−0.290*	[−0.512, −0.068]	−0.447*	[−0.676, −0.218]	−0.156	[−0.358, 0.046]
Age	−0.007	[−0.025, 0.011]	−0.016	[−0.034, 0.002]	−0.009	[−0.025, 0.007]
Grade	0.034	[−0.033, 0.101]	0.049	[−0.022, 0.120]	0.015	[−0.046, 0.076]
News exposure	−0.052	[−0.117, 0.013]	−0.012	[−0.079, 0.055]	0.040	[−0.019, 0.099]
News interest	0.363*	[0.236, 0.490]	0.228*	[0.097, 0.359]	−0.135*	[−0.250, −0.020]
News trust	0.174*	[0.061, 0.287]	0.055	[−0.062, 0.172]	−0.120*	[−0.221, −0.019]

*n* = 138 (2 participants with missing news exposure data were excluded listwise). Gender coded 0 = Female, 1 = Male. Diff = AI-generated news quality minus human-written news quality; a significant coefficient in this column indicates a significant interaction between the corresponding predictor and Authorship.

*p < 0.05. Human model: *R*^2^ = 0.303, *F*(6, 131) = 9.47, *p* < 0.001. AI model: *R*^2^ = 0.219, *F*(6, 131) = 6.11, *p* < 0.001. Diff model: *R*^2^ = 0.110, *F*(6, 131) = 2.69, *p* = 0.017.

This pattern is noteworthy because all evaluations were conducted under blind conditions, and participants were unaware of whether a given article was AI-generated or human-written; the observed asymmetry therefore cannot be attributed to readers’ awareness of content origin. One possible interpretation is that human-written news possesses greater stylistic diversity and individual authorial variation, leaving more room for personal media orientations to influence evaluations, whereas AI-generated news may be evaluated more uniformly across readers regardless of their individual news interest or trust. However, as noted above, the present textual analysis found that AI-generated articles were not lexically more uniform than human-written articles; the mechanism underlying this attenuation therefore remains unclear and may involve structural or stylistic properties of AI-generated text other than lexical diversity.

From a cultural psychological perspective, this pattern may be related to the nature of interdependent self-construal prevalent in both Chinese and Vietnamese cultural contexts. Individuals with an interdependent self-construal tend to base their judgments on situational cues and perceived social consensus rather than on stable internal traits or personal dispositions (Markus and Kitayama, 1991). AI-generated news, as a depersonalized textual form stripped of individual authorial voice and culturally situated perspective, may align with this situation-based evaluative orientation in ways that are not yet well understood: without a human author to anchor relational or hierarchical judgments, readers may respond primarily to other textual properties that were not directly examined here. This cultural dynamic may help explain why individual-level variables such as news interest and trust, which typically serve as heuristic anchors when evaluating the intentions and credibility of a human author, appear to lose predictive power when the author is an AI system rather than a culturally situated human communicator.

## 5 Conclusion

This study examined how journalism students in China and Vietnam evaluated the quality of AI-generated news and human-written news under blind conditions, using a mixed experimental design covering four news categories and seven quality dimensions. Participants generally rated AI-generated news higher overall, particularly on structural dimensions including perceived headline relevance, coverage adequacy, readability, and punctuation and sentence structure. Human-written news maintained parity with AI-generated news on perceived factual accuracy and source usage. A mixed-design ANOVA further showed that this AI advantage was not uniform: it was more pronounced in the Nature and Society categories than in Health and Finance, and was significantly larger among Chinese than Vietnamese participants, while the three-way Country × Authorship × Topic interaction was not significant, suggesting that this topic-specific pattern did not differ substantially between the two countries.

Cross-national comparisons revealed that Chinese and Vietnamese students differed significantly in their evaluations of human-written news, with Vietnamese students assigning higher scores, while evaluations of AI-generated news were comparatively consistent across both groups. Divergence in human-written news evaluations may reflect the distinct media cultures and journalistic traditions of the two societies, while convergence in AI-generated news evaluations has been proposed in the literature to reflect the homogenizing tendencies of large language models; however, the present textual analysis found that AI-generated articles were not lexically more uniform than human-written articles, so the specific mechanism underlying this convergence remains an open question. News interest and news trust predicted evaluations of human-written news more strongly than evaluations of AI-generated news, a pattern confirmed by direct interaction tests; gender, by contrast, predicted evaluations of both news types without a significant authorship-specific asymmetry. These individual-level patterns are discussed in relation to the situation-based evaluative orientation characteristic of interdependent self-construal cultures, though this construct was not directly measured.

This study has several limitations. A central limitation concerns the stimulus materials used: each Country × Topic × Authorship cell was represented by only a single article pair, so the statistical models could not separate variance attributable to the specific stimulus materials from variance attributable to Country, Topic, or Authorship. Relatedly, Country was inevitably confounded with language, media outlet, and the specific news events selected for each nation, in part because China and Vietnam differ substantially in the news events, policy environments, and regulatory contexts that shape what constitutes representative news coverage; no single set of articles could be presented in identical form across the two samples while still reflecting authentic, contextually representative journalism in each country. The cross-national and topic-specific patterns reported here should therefore be interpreted as findings specific to the particular stimulus materials employed, rather than as fully generalizable properties of Chinese and Vietnamese news audiences. Further, although lexical (Jaccard) overlap analysis indicated that the AI-generated articles were not verbatim reproductions of their human-written counterparts, this measure reflects wording overlap only and does not establish content equivalence; differences in the specific factual propositions, contextual details, or narrative organization between the two versions of each article were not independently verified, and may represent a further potential confound when interpreting the authorship effect, alongside the objective differences in length, sentence structure, lexical diversity, and source-attribution markers documented in the Materials section. A further limitation concerns the theoretical constructs invoked to interpret the cross-national and individual-level findings: explanations drawing on power distance, self-construal, long-term orientation, techno-optimism, and the authority heuristic relied on country membership as a proxy for these constructs, informed by national-level scores from existing cross-cultural frameworks (e.g., Hofstede et al., 2010); none of these constructs, nor AI literacy, AI familiarity, or institutional trust, were directly measured among individual participants. Similarly, cultural or textual convergence, including possible convergence in narrative structure, framing, or rhetorical style, was not directly measured; the present study assessed convergence only in participants’ quality evaluations, and any broader cultural or textual convergence remains a theoretical extrapolation beyond what these evaluative data can directly establish. These interpretations should accordingly be understood as theoretically motivated accounts compatible with the observed patterns rather than empirically verified mechanisms. In addition, the sample was drawn from a limited number of institutions in China and Vietnam and may not fully represent journalism students in either country; only one AI model (DeepSeek) was used, which limits generalizability across generative AI systems; and the study materials consisted solely of news reports, a genre that prioritizes structural clarity over narrative depth and may have amplified AI’s structural advantages, consistent with prior evidence that AI-generated text tends to be more analytical but lower in readability than human-written text (Markowitz et al., 2023). Finally, perceived factual accuracy and source usage, as reported in Table 6, reflect participants’ subjective judgments rather than independently verified textual properties, and presentation order was counterbalanced across topic categories rather than randomized at the individual level, which may not fully rule out order effects specific to individual participants.

Future research should address these limitations in several ways. Employing multiple independent article pairs per condition, ideally pretested for content and difficulty equivalence and translated using validated back-translation procedures, would allow stimulus-level variability to be modeled explicitly and separated from the effects of substantive interest. Incorporating validated individual-level measures of power distance, self-construal, long-term orientation, techno-optimism, AI literacy, AI familiarity, and institutional trust would permit the cultural-psychological mechanisms proposed here to be tested directly rather than inferred from country membership. Future studies would also benefit from more broadly representative samples, comparisons across multiple AI models and text genres, independent verification of factual accuracy and source reliability by trained coders or subject-matter experts, and individually randomized presentation order. Beyond these methodological refinements, the comparative framework could be extended to additional cultural contexts beyond East Asia and Southeast Asia, and future work might examine how cultural variables such as power distance and self-construal interact with explicit AI authorship disclosure. As generative AI continues to reshape media landscapes globally, understanding how culturally grounded psychological processes mediate audience responses to AI-generated content remains a pressing area of inquiry for cross-cultural psychology.

## Data Availability

The raw data supporting the conclusions of this article will be made available by the authors, without undue reservation.

## Footnotes

1
https://www.bjnews.com.cn/site/about

2
https://vnexpress.net/
