# Correction: Perceived quality of AI-generated and human-written news: a cross-national experimental study among journalism students in China and Vietnam

**DOI:** 10.3389/fpsyg.2026.2015504

**Published:** 2026-10-27

**Authors:** Mingyi Yang, Yaqi Deng, Yuting Cheng

**Affiliations:** School of Journalism and Communication, Guangxi University, Nanning, China

**Keywords:** AI-generated news, cross-cultural comparison, generative artificial intelligence, news quality evaluation, power distance

In the published article, an incorrect funder name and incorrect grant number was provided. The correct funder is Guangxi Science and Technology Program and the correct grant number is GKAD23026246. The correct **Funding** statement reads:

The author(s) declared that financial support was received for this work and/or its publication. This research was funded by Guangxi Science and Technology Program (No. GKAD23026246). The APC was funded by the Science and Technology Department of Guangxi Zhuang Autonomous Region.

In addition, the reference for [Blanca et al., 2017] was erroneously written as [Blanca, M. J., Alarcón, R., Arnau, J., Bono, R., and Bendayan, R. (2017). Non-normal data: Is ANOVA still a valid option? *Psicothema* 29, 552–557.].

It should be [Blanca, M. J., Alarcón, R., Arnau, J., Bono, R., and Bendayan, R. (2017). Non-normal data: Is ANOVA still a valid option? *Psicothema* 29, 552–557. doi: 10.7334/psicothema2016.383].

Further, the reference for the in-text citation in the footnote of Table 3 was erroneously written as “following Steiger (2024)”. It should be “following Steiger (2004)”.

Also, the Vietnamese diacritical characters were rendered incorrectly at Section 2.3 (**Materials**), in the paragraph discussing Vietnamese word segmentation.

The erroneous published text reads: [“thu'o'ng mại”]

The correct text should read: [“thúóng mại”].

The original version of this article has been updated.

