# Supplementary material for: Perceived quality of AI-generated and human-written news: a cross-national experimental study among journalism students in China and Vietnam
Source: Front Psychol. 2026 Sep 23;17:1886638. doi: 10.3389/fpsyg.2026.1886638 (PMC13642218; doi:10.3389/fpsyg.2026.1886638)
Supplement: Supplementary file 1 [file Table_1.docx]

**Supplementary Data Sheet 1**

**Questionnaire Instrument (Chinese and Vietnamese Versions)**

*This supplementary material presents the questionnaire instrument used in the study, in Chinese and Vietnamese. The questionnaire was administered online via Wenjuanxing (问卷星), a Chinese online survey platform comparable to Qualtrics or SurveyMonkey; the text below reflects the item content and structure as presented to participants. News stimulus materials are omitted due to third-party copyright considerations; an illustrative example of the stimulus format is provided in Figure 2 of the main text.*

**中文版：大学生对网络新闻质量的评价调查**

欢迎参与本次调查。本问卷旨在了解受众对网络新闻内容质量的评价方式。我们是一组研究团队，正在探索新闻报道质量的评估与规范。为此，我们需要了解不同受众对在线新闻的阅读与判断情况。

本问卷为匿名填写，所有信息将严格保密，仅用于科研研究。完成问卷预计耗时约 15 分钟。

*提示：若您在阅读新闻材料时文字显示较小，可点击新闻图片后放大页面，方便清晰阅读。*

**知情同意**

**1. 请您确认以下内容后，再进行后续操作：
您自愿参与本次研究；您已年满 18 周岁，具备参与本研究的主体资格；您知晓在研究过程中，可随时以任何理由无条件退出，且不会因此受到任何不利影响。**

○ 同意 / ○ 不同意（跳至问卷末尾，提交答卷）

**一、新闻消费习惯**

**2. 你日常使用互联网的频率如何（不限用途、设备及使用地点）？**

每周不足1次 / 每周1次 / 每周2-3天 / 每周4-6天 / 每天1次 / 每天2-5次 / 每天6-10次 / 每天10次以上 / 不确定

**3. 通常情况下，你接触新闻的频率如何？（注：此处“新闻”涵盖国内、国际、地区/地方新闻及其他时事内容，接触渠道包括广播、电视、报纸、网络等各类平台。）**

每周不足1次 / 每周1次 / 每周2-3天 / 每周4-6天 / 每天1次 / 每天2-5次 / 每天6-10次 / 每天10次以上 / 不确定

**4. 你对新闻的感兴趣程度如何？**

不感兴趣 / 不太感兴趣 / 有一定兴趣 / 比较感兴趣 / 非常感兴趣 / 不确定

**5. 我认为在大多数情况下，我可以信任大部分新闻。**

完全同意 / 同意 / 中立 / 不同意 / 完全不同意

**二、新闻质量评价^[1]^**

**6. 新闻易于阅读和理解**

|  | 非常不同意 | 不同意 | 比较不同意 | 中立 | 比较同意 | 同意 | 非常同意 |
| --- | --- | --- | --- | --- | --- | --- | --- |
| **新闻1** | ○ | ○ | ○ | ○ | ○ | ○ | ○ |
| **新闻2** | ○ | ○ | ○ | ○ | ○ | ○ | ○ |

**7. 新闻以一致、准确且有序的方式呈现事实**

|  | 非常不同意 | 不同意 | 比较不同意 | 中立 | 比较同意 | 同意 | 非常同意 |
| --- | --- | --- | --- | --- | --- | --- | --- |
| **新闻1** | ○ | ○ | ○ | ○ | ○ | ○ | ○ |
| **新闻2** | ○ | ○ | ○ | ○ | ○ | ○ | ○ |

**8. 新闻的标题很好地反映了其主要内容**

|  | 非常不同意 | 不同意 | 比较不同意 | 中立 | 比较同意 | 同意 | 非常同意 |
| --- | --- | --- | --- | --- | --- | --- | --- |
| **新闻1** | ○ | ○ | ○ | ○ | ○ | ○ | ○ |
| **新闻2** | ○ | ○ | ○ | ○ | ○ | ○ | ○ |

**9. 新闻对主题进行了详细的报道，提供了足够的信息以理解整个事件**

|  | 非常不同意 | 不同意 | 比较不同意 | 中立 | 比较同意 | 同意 | 非常同意 |
| --- | --- | --- | --- | --- | --- | --- | --- |
| **新闻1** | ○ | ○ | ○ | ○ | ○ | ○ | ○ |
| **新闻2** | ○ | ○ | ○ | ○ | ○ | ○ | ○ |

**10. 新闻以充分且平衡的方式呈现消息来源**

|  | 非常不同意 | 不同意 | 比较不同意 | 中立 | 比较同意 | 同意 | 非常同意 |
| --- | --- | --- | --- | --- | --- | --- | --- |
| **新闻1** | ○ | ○ | ○ | ○ | ○ | ○ | ○ |
| **新闻2** | ○ | ○ | ○ | ○ | ○ | ○ | ○ |

**11. 新闻使用了可能让读者困惑的复杂语言（反向计分）**

|  | 非常不同意 | 不同意 | 比较不同意 | 中立 | 比较同意 | 同意 | 非常同意 |
| --- | --- | --- | --- | --- | --- | --- | --- |
| **新闻1** | ○ | ○ | ○ | ○ | ○ | ○ | ○ |
| **新闻2** | ○ | ○ | ○ | ○ | ○ | ○ | ○ |

**12. 新闻中明显体现出标点符号的恰当使用和清晰句子的构建**

|  | 非常不同意 | 不同意 | 比较不同意 | 中立 | 比较同意 | 同意 | 非常同意 |
| --- | --- | --- | --- | --- | --- | --- | --- |
| **新闻1** | ○ | ○ | ○ | ○ | ○ | ○ | ○ |
| **新闻2** | ○ | ○ | ○ | ○ | ○ | ○ | ○ |

**三、背景信息**

**34. 你的年龄是（请输入数字，如20）：**

______________

**35. 你的性别是：**

男 / 女

**36. 你的年级是：**

大一 / 大二 / 大三 / 大四 / 研一 / 研二 / 研三 / 博士

**37. 你的专业是（如新闻与传播学专硕、新闻传播学学硕、新闻学专业、广告学专业、播音与主持专业等）：**

______________

**38. 你的学校是：**

______________

**Vietnamese Version: Đánh giá của độc giả về khảo sát chất lượng tin tức trực tuyến**

Chào mừng bạn đến với khảo sát này. Bảng câu hỏi này nhằm mục đích tìm hiểu cách độc giả đánh giá chất lượng nội dung tin tức trực tuyến. Chúng tôi là một nhóm nghiên cứu đang tìm hiểu việc đánh giá và chuẩn hóa chất lượng đưa tin. Để đạt được mục đích này, chúng tôi cần hiểu cách các độc giả khác nhau đọc và đánh giá tin tức trực tuyến.

Bảng câu hỏi này được ẩn danh, mọi thông tin sẽ được bảo mật tuyệt đối và chỉ được sử dụng cho mục đích nghiên cứu. Việc hoàn thành bảng câu hỏi dự kiến mất khoảng 15 phút.

*Mẹo: Nếu chữ hiển thị nhỏ khi đọc tin tức, bạn có thể nhấp vào hình ảnh tin tức để phóng to trang và đọc rõ hơn.*

**Informed Consent**

**1. Câu trả lời của bạn rất quan trọng. Bằng cách nhấp vào nút bên dưới, bạn xác nhận: Bạn tự nguyện tham gia nghiên cứu này; Bạn từ 18 tuổi trở lên; Bạn có thể rút khỏi nghiên cứu này bất cứ lúc nào vì bất kỳ lý do gì.**

○ Đồng ý / ○ Không đồng ý

**I. Thói quen tiêu thụ tin tức**

**2. Tần suất bạn sử dụng Internet trong sinh hoạt hằng ngày như thế nào? (không giới hạn mục đích, thiết bị và địa điểm sử dụng)**

Ít hơn 1 lần/tuần / 1 lần/tuần / 2–3 ngày/tuần / 4–6 ngày/tuần / 1 lần/ngày / 2–5 lần/ngày / 6–10 lần/ngày / Trên 10 lần/ngày / Không chắc chắn

**3. Thông thường, tần suất bạn tiếp xúc với tin tức là bao nhiêu? (Ghi chú: “Tin tức” ở đây bao gồm tin tức trong nước, quốc tế, địa phương; các kênh tiếp xúc bao gồm radio, TV, báo in, internet và các nền tảng khác)**

Ít hơn 1 lần/tuần / 1 lần/tuần / 2–3 ngày/tuần / 4–6 ngày/tuần / 1 lần/ngày / 2–5 lần/ngày / 6–10 lần/ngày / Trên 10 lần/ngày / Không chắc chắn

**4. Mức độ quan tâm đến tin tức của bạn như thế nào?**

Không quan tâm / Ít quan tâm / Có chút quan tâm / Khá quan tâm / Rất quan tâm / Không chắc chắn

**5. Tôi cho rằng trong phần lớn trường hợp, tôi có thể tin tưởng phần lớn các bản tin.**

Hoàn toàn đồng ý / Đồng ý / Trung lập / Không đồng ý / Hoàn toàn không đồng ý

**II. Đánh giá chất lượng tin tức^[2]^**

**6. Tin tức dễ đọc và dễ hiểu**

|  | Hoàn toàn không đồng ý | Không đồng ý | Khá không đồng ý | Trung lập | Khá đồng ý | Đồng ý | Hoàn toàn đồng ý |
| --- | --- | --- | --- | --- | --- | --- | --- |
| **Tin tức 1** | ○ | ○ | ○ | ○ | ○ | ○ | ○ |
| **Tin tức 2** | ○ | ○ | ○ | ○ | ○ | ○ | ○ |

**7. Tin tức trình bày sự kiện một cách nhất quán, chính xác và có trật tự**

|  | Hoàn toàn không đồng ý | Không đồng ý | Khá không đồng ý | Trung lập | Khá đồng ý | Đồng ý | Hoàn toàn đồng ý |
| --- | --- | --- | --- | --- | --- | --- | --- |
| **Tin tức 1** | ○ | ○ | ○ | ○ | ○ | ○ | ○ |
| **Tin tức 2** | ○ | ○ | ○ | ○ | ○ | ○ | ○ |

**8. Tiêu đề của tin tức phản ánh tốt nội dung chính**

|  | Hoàn toàn không đồng ý | Không đồng ý | Khá không đồng ý | Trung lập | Khá đồng ý | Đồng ý | Hoàn toàn đồng ý |
| --- | --- | --- | --- | --- | --- | --- | --- |
| **Tin tức 1** | ○ | ○ | ○ | ○ | ○ | ○ | ○ |
| **Tin tức 2** | ○ | ○ | ○ | ○ | ○ | ○ | ○ |

**9. Tin tức đưa ra báo cáo chi tiết về chủ đề, cung cấp đủ thông tin để hiểu toàn bộ sự kiện**

|  | Hoàn toàn không đồng ý | Không đồng ý | Khá không đồng ý | Trung lập | Khá đồng ý | Đồng ý | Hoàn toàn đồng ý |
| --- | --- | --- | --- | --- | --- | --- | --- |
| **Tin tức 1** | ○ | ○ | ○ | ○ | ○ | ○ | ○ |
| **Tin tức 2** | ○ | ○ | ○ | ○ | ○ | ○ | ○ |

**10. Tin tức trình bày nguồn thông tin một cách đầy đủ và cân bằng**

|  | Hoàn toàn không đồng ý | Không đồng ý | Khá không đồng ý | Trung lập | Khá đồng ý | Đồng ý | Hoàn toàn đồng ý |
| --- | --- | --- | --- | --- | --- | --- | --- |
| **Tin tức 1** | ○ | ○ | ○ | ○ | ○ | ○ | ○ |
| **Tin tức 2** | ○ | ○ | ○ | ○ | ○ | ○ | ○ |

**11. Tin tức sử dụng ngôn ngữ phức tạp có thể gây nhầm lẫn cho người đọc (thang đo ngược)**

|  | Hoàn toàn không đồng ý | Không đồng ý | Khá không đồng ý | Trung lập | Khá đồng ý | Đồng ý | Hoàn toàn đồng ý |
| --- | --- | --- | --- | --- | --- | --- | --- |
| **Tin tức 1** | ○ | ○ | ○ | ○ | ○ | ○ | ○ |
| **Tin tức 2** | ○ | ○ | ○ | ○ | ○ | ○ | ○ |

**12. Tin tức thể hiện rõ việc sử dụng dấu câu phù hợp và xây dựng câu rõ ràng**

|  | Hoàn toàn không đồng ý | Không đồng ý | Khá không đồng ý | Trung lập | Khá đồng ý | Đồng ý | Hoàn toàn đồng ý |
| --- | --- | --- | --- | --- | --- | --- | --- |
| **Tin tức 1** | ○ | ○ | ○ | ○ | ○ | ○ | ○ |
| **Tin tức 2** | ○ | ○ | ○ | ○ | ○ | ○ | ○ |

**III. Thông tin nhân khẩu học xã hội**

**34. Tuổi của bạn là (nhập một số, chẳng hạn như 20):**

______________

**35. Giới tính của bạn là:**

Nam / Nữ / Khác

**36. Trình độ học vấn của bạn:**

Đại học năm 1 / Đại học năm 2 / Đại học năm 3 / Đại học năm 4 / Thạc sĩ năm 1 / Thạc sĩ năm 2 / Thạc sĩ năm 3 / Tiến sĩ

**37. Chuyên ngành của bạn là (ví dụ: Thạc sĩ Báo chí và Truyền thông, Thạc sĩ Khoa học Báo chí và Truyền thông, Báo chí, Quảng cáo, Phát thanh và Dẫn chương trình...):**

______________

**38. Trường học của bạn là:**

______________

1. This section was administered four times, once for each news topic category (Nature, Health, Society, and Finance), following an identical format each time. In each administration, participants viewed one human-written article and one AI-generated article on the same topic and rated both using the seven items below. News stimulus materials are not reproduced here due to third-party copyright restrictions; an illustrative example is provided in Figure 2 of the main text.
2. This section was administered four times, once for each news topic category (Nature, Health, Society, and Finance), following an identical format each time. In each administration, participants viewed one human-written article and one AI-generated article on the same topic and rated both using the seven items below. News stimulus materials are not reproduced here due to third-party copyright restrictions; an illustrative example is provided in Figure 2 of the main text.
