# Supplementary material for: Perceived quality of AI-generated and human-written news: a cross-national experimental study among journalism students in China and Vietnam
Source: Front Psychol. 2026 Sep 23;17:1886638. doi: 10.3389/fpsyg.2026.1886638 (PMC13642218; doi:10.3389/fpsyg.2026.1886638)
Supplement: Supplementary file 2 [file Table_2.docx]

Supplementary Table 1. Objective Textual Features of Human-Written and AI-Generated Articles, by Country, Topic, and Authorship.

| Country | Topic | Authorship | Headline Length | Article Length | N Sentences | Avg. Sentence Length | TTR | MTLD | Source Markers |
| --- | --- | --- | --- | --- | --- | --- | --- | --- | --- |
| China | Nature | Human-Written | 12 chars | 162 chars | 3 | 54.0 chars | 0.725 | 70.2 | 1 |
| China | Nature | AI-Generated | 18 chars | 271 chars | 6 | 45.2 chars | 0.740 | 113.1 | 1 |
| China | Health | Human-Written | 23 chars | 200 chars | 2 | 100.0 chars | 0.617 | 55.6 | 3 |
| China | Health | AI-Generated | 27 chars | 206 chars | 5 | 41.2 chars | 0.792 | 129.0 | 0 |
| China | Society | Human-Written | 20 chars | 101 chars | 2 | 50.5 chars | 0.830 | 77.3 | 0 |
| China | Society | AI-Generated | 21 chars | 112 chars | 4 | 28.0 chars | 0.885 | 126.2 | 0 |
| China | Finance | Human-Written | 17 chars | 133 chars | 3 | 44.3 chars | 0.857 | 109.8 | 2 |
| China | Finance | AI-Generated | 23 chars | 197 chars | 4 | 49.2 chars | 0.758 | 105.4 | 0 |
| Vietnam | Nature | Human-Written | 7 words | 168 words | 9 | 18.7 words | 0.638 | 140.7 | 1 |
| Vietnam | Nature | AI-Generated | 13 words | 144 words | 6 | 24.0 words | 0.700 | 193.0 | 1 |
| Vietnam | Health | Human-Written | 9 words | 232 words | 12 | 19.3 words | 0.587 | 127.1 | 1 |
| Vietnam | Health | AI-Generated | 9 words | 226 words | 11 | 20.5 words | 0.568 | 154.5 | 1 |
| Vietnam | Society | Human-Written | 7 words | 126 words | 8 | 15.8 words | 0.675 | 141.5 | 0 |
| Vietnam | Society | AI-Generated | 8 words | 78 words | 5 | 15.6 words | 0.873 | 242.0 | 0 |
| Vietnam | Finance | Human-Written | 7 words | 276 words | 10 | 27.6 words | 0.509 | 122.3 | 7 |
| Vietnam | Finance | AI-Generated | 10 words | 237 words | 10 | 23.7 words | 0.541 | 143.5 | 3 |

Note. TTR = type-token ratio; MTLD = Measure of Textual Lexical Diversity. Chinese text was segmented using jieba; Vietnamese text was segmented using underthesea. Source Markers = count of explicit source-attribution expressions (e.g., “according to,” “officials said,” “announced”) per article. Headline and article length are reported in characters for Chinese and words for Vietnamese, reflecting the two writing systems.

Supplementary Table 2. Lexical (Jaccard) Overlap Between Human-Written and AI-Generated Article Pairs, by Country and Topic.

| Country | Topic | Jaccard Overlap | Shared Tokens | Human-Only Tokens | AI-Only Tokens |
| --- | --- | --- | --- | --- | --- |
| China | Nature | 0.254 | 29 | 22 | 63 |
| China | Health | 0.301 | 31 | 27 | 45 |
| China | Society | 0.349 | 22 | 17 | 24 |
| China | Finance | 0.427 | 35 | 13 | 34 |
| Vietnam | Nature | 0.297 | 52 | 64 | 59 |
| Vietnam | Health | 0.302 | 71 | 86 | 78 |
| Vietnam | Society | 0.288 | 36 | 56 | 33 |
| Vietnam | Finance | 0.310 | 74 | 86 | 79 |

Note. Jaccard overlap = (shared tokens) / (shared + human-only + AI-only tokens), computed on segmented word/character tokens after removing punctuation. Low-to-moderate overlap values indicate that AI-generated articles substantially reworded rather than reproduced the corresponding human-written articles.

Supplementary Table 3. Generation Details and Complete Prompts Used to Produce the Eight AI-Generated Stimulus Articles.

| Country | Topic | Generation Date | Prompt Language | Complete Prompt (Original Text) |
| --- | --- | --- | --- | --- |
| China | Nature | 2025-11-06 | Chinese | What（事件）：2025 年最大 “超级月亮” 现身夜空  Why（原因）：11 月 6 日月球过近地点时距地球约 35.7 万千米，是 2025 年所有满月中与地球最近的一次，故 5 日晚的月亮为年度最大满月  When（时间）：11 月 5 日 21 时 19 分前后月亮最完满，11 月 6 日 6 时 27 分月球过近地点  Who（相关方）：天文学家（提供相关预测信息）  Where（地点）：北京及所有夜空（无具体限定地域） 根据提供的 5W 新闻要素撰写一条包含新闻标题的新闻报道，150 字左右 |
| China | Health | 2025-11-06 | Chinese | What（事件）：2025年国家医保药品目录谈判竞价及商保创新药目录价格协商顺利结束 Why（原因）：优化医保药品目录，提升患者用药可及性，完善多层次医疗保障体系 When（时间）：2025年10月30日至11月3日谈判；12月第一个周末发布目录，明年1月1日实施 Who（相关方）：国家医保局、120家内外资参与企业 Where（地点）：北京全国人大会议中心谈判；广州线上线下同步发布目录 根据提供的 5W 新闻要素撰写一条包含新闻标题的新闻报道，200 字左右。 |
| China | Society | 2025-11-07 | Chinese | What（事件）：夫妻登山赏秋，妻子下山时迷路被困  Why（原因）：下山时丈夫先行去开车，妻子单独行动后迷失方向  When（时间）：2025 年 11 月 1 日傍晚（18 时 27 分消防出发，当晚护送下山）  Who（人物）：迷路女子、其丈夫、北京市门头沟区龙泉消防救援站救援人员  Where（地点）：北京市门头沟区山区 根据提供的 5W 新闻要素撰写一条包含新闻标题的新闻报道，100 字左右。 |
| China | Finance | 2025-11-06 | Chinese | What（事件）：“升级版”中欧出口管制对话磋商举行 Why（原因）：落实中欧领导人经贸共识，沟通出口管制领域关切，促进产业链供应链稳定 When（时间）：2025年10月31日至11月1日 Who（相关方）：商务部安全与管制局江前良局长、欧委会贸易总司雷东内副总司长 Where（地点）：比利时布鲁塞尔 根据提供的 5W 新闻要素撰写一条包含新闻标题的新闻报道，150 字左右。 |
| Vietnam | Nature | 2025-12-05 | Vietnamese (5W labels in English) | Who: Nhân vật chính: Ông Đinh Đức Dung, 63 tuổi, ngụ tại xã Tân Kỳ. Cơ quan thực hiện: Hạt kiểm lâm huyện Tân Kỳ phối hợp với chính quyền xã Tân Kỳ. What: Một cá thể chim Hồng hoàng quý hiếm bay vào nhà dân, sau đó được bàn giao cho kiểm lâm và thả về tự nhiên thành công. When: Thời điểm phát hiện: Ngày 3 tháng 10. Thời điểm thả về rừng: Ngày 8 tháng 10. Where: Địa điểm phát hiện: Khối 1, xã Tân Kỳ, huyện Tân Kỳ, tỉnh Nghệ An. Địa điểm thả về: Khu vực rừng trên địa bàn huyện Tân Kỳ. Why: Cá thể chim này được xác định là Hồng hoàng, thuộc nhóm IB - loài nguy cấp, quý hiếm, bị nghiêm cấm khai thác, sử dụng vì mục đích thương mại. Việc thả về rừng nhằm bảo tồn loài này. Dựa trên thông tin tin tức 5W này, hãy viết một bài tin tiếng Việt khoảng 200 chữ kèm tiêu đề. |
| Vietnam | Health | 2025-12-05 | Vietnamese (5W labels in English) | Who: Cục Quản lý Dược và Công ty Medipharco What: Cục Quản lý Dược yêu cầu Công ty Medipharco thu hồi thuốc giảm đau hạ sốt Padobaby dạng bột trên toàn quốc When: Ngày 25 tháng 11 tiến hành kiểm tra và đưa ra quyết định, hoàn thành trong 30 ngày Where: Trên toàn quốc Why: Lô sản phẩm có số hiệu 110224, hạn sử dụng đến tháng 2 năm 2027, chứa chất bảo quản methylparaben với hàm lượng không đạt tiêu chuẩn, thuộc vi phạm cấp ba. Dựa trên thông tin tin tức 5W này, hãy viết một bài tin tiếng Việt khoảng 300 chữ kèm tiêu đề. |
| Vietnam | Society | 2025-12-05 | Vietnamese (5W labels in English) | Who: Tài xế Nguyễn Xuân Hải (33 tuổi) What: Xe tải đâm vào dải phân cách rồi lật, gây tắc nghẽn giao thông, tài xế không bị thương. When: Chiều 3/12 (khoảng 15 giờ). Where: Trên đoạn đường Núi Cù Hin, qua xã Cam Lâm, hướng từ Nha Trang đến sân bay Cam Ranh, tỉnh Khánh Hòa. Why: Khi xảy ra tai nạn trời mưa, đoạn đường nhiều khúc cua và mật độ xe cao. Dựa trên thông tin tin tức 5W này, hãy viết một bài tin tiếng Việt khoảng 150 chữ kèm tiêu đề. |
| Vietnam | Finance | 2025-12-05 | Vietnamese (5W labels in English) | Who: Bộ Ngoại giao Việt Nam, Văn phòng Đại diện Thương mại Hoa Kỳ - USTR What: Hoa Kỳ quyết định không áp dụng các biện pháp điều chỉnh thương mại đối với Việt Nam, Việt Nam bày tỏ hoan nghênh When: Công bố ngày 23 tháng 7 Where: Quan hệ song phương Việt Nam - Hoa Kỳ Why: Đây là quyết định của USTR sau cuộc điều tra về chính sách tiền tệ của Việt Nam, là kết quả của đối thoại xây dựng và thiện chí giữa hai bên, dựa trên thỏa thuận về cam kết quản lý tỷ giá giữa Ngân hàng Nhà nước Việt Nam và Bộ Tài chính Mỹ. Dựa trên thông tin tin tức 5W này, hãy viết một bài tin tiếng Việt khoảng 400 chữ kèm tiêu đề. |

Note. All prompts were entered manually via the DeepSeek web/app interface (DeepSeek-V3.1), not via API. For all 16 generations: the “Deep Thinking” (reasoning) and web search functions were both disabled; each prompt was submitted once, yielding a single output with no candidate selection; no system prompt was set beyond the platform's default; generation parameters (e.g., temperature, top-p) were not user-configurable in this interface and therefore followed the platform's internal defaults; and no post-generation manual editing was applied to any AI output. Target word counts were calibrated for each article to approximate the length of the corresponding human-written original (see Materials).
